# A Study of Free-Form Shape Rationalization Using Biomimicry as Inspiration

**DOI:** 10.3390/polym15112466

**Published:** 2023-05-26

**Authors:** Gaurab Sundar Dutta, Dieter Meiners, Nina Merkert

**Affiliations:** 1 Institute of Polymer Materials and Plastics Technology, Clausthal University of Technology, Agricolastr. 6, 38678 Clausthal, Germany; dieter.meiners@tu-clausthal.de; 2 Institute of Applied Mechanics, Clausthal University of Technology, Arnold-Sommerfeld-Str.6, Room 310, 38678 Clausthal, Germany; nina.merkert@tu-clausthal.de

**Keywords:** shape rationalization, biological forms, free-form geometry, visual programming, parametric modelling, fiber composite, digital fabrication

## Abstract

Bridging the gap between the material and geometrical aspects of a structure is critical in lightweight construction. Throughout the history of structural development, shape rationalization has been of prime focus for designers and architects, with biological forms being a major source of inspiration. In this work, an attempt is made to integrate different phases of design, construction, and fabrication under a single framework of parametric modeling with the help of visual programming. The idea is to offer a novel free-form shape rationalization process that can be realized with unidirectional materials. Taking inspiration from the growth of a plant, we established a relationship between form and force, which can be translated into different shapes using mathematical operators. Different prototypes of generated shapes were constructed using a combination of existing manufacturing processes to test the validity of the concept in both isotropic and anisotropic material domains. Moreover, for each material/manufacturing combination, generated geometrical shapes were compared with other equivalent and more conventional geometrical constructions, with compressive load-test results being the qualitative measure for each use case. Eventually, a 6-axis robot emulator was integrated with the setup, and corresponding adjustments were made such that a true free-form geometry could be visualized in a 3D space, thus closing the loop of digital fabrication.

## 1. Introduction

### 1.1. Early Motivation

Design, depending on the area of application, differs in its definition. In structural engineering, it is the process of investigating a given structure for stability and strength; under the prescribed loading scenario. On the other hand, design as the trade of architecture is mostly a shape rationalization process. In this context, design can be considered as a method to search, which allows the designer to realize, alter and correlate different independent variables involved in a complex process. The lack of complete problem definition and constraints makes the problem ill-poised. In recent years, advancements in computational tools have allowed architects to create increasingly complex shapes, thus bringing the issue of ill-poised problems more and more into the spotlight.

In our previous paper [1], we discussed how design as a search process could yield a unique range of generative solutions for simple problems. In this paper, we will elaborate the idea further by first looking into the historical development in the shape rationalization process and how various technological developments impacted this approach. Later, as a continuation of our previous work, we will look into the process of evolution of more complex shapes from basic curves and will try to estimate the merit of the solutions against imposed requirements or boundary conditions based on their performances. Finally, we will address how these solutions can be integrated with the digital fabrication process.

### 1.2. Brief History of Shape Rationalization

Construction of a complex shape is a rigorous process involving creativity, technological solutions, and economic and ecological aspects altogether. Hence it is wise to introduce fabrication constraints into the design process itself in order to address manufacturing issues at the very beginning. This process in modern architecture and engineering is referred to as shape rationalization or constructive geometry. Shape rationalization has been widely used for decades and found its root in the eighteenth century among the fields such as stereotomy. Stereotomy typically refers to the art of cutting solids precisely to assemble them into complex shapes [2].

Until the 16th century, during the ages of Renaissance Humanism, the designer of a building was also responsible for the realization of his designs, making the practicalities of construction an integral constituent in any design decision. This deep connection between design, structure, materiality, and realization is apparent in masterpieces such as the Pyramids, the Pantheon, or any of the great Gothic cathedrals, where contemporary building capabilities were pushed to their limit. In fact, the definition of modulus evolved from the architecture field (to define a unitary representative part of the whole construction), which later translated into other fields [3]. It is only with the separation between the architect and the builder in the later century that the disciplines of structure and construction split from the stylistic, aesthetic craft of the architect.

Highly regarded as the pioneer of this field, Gaspard Monge, in the 17th century, brought the shape realization process of a structure back into the mainstream. He described ‘constructive geometry’ as an art having two main objectives. “…The first is to obtain an exact representation on two dimensional drawings of three-dimensional objects that require rigorous definition... The second is to deduce from the exact description of bodies, all that necessarily follows from their shape and their respective positions” [4].

In the coming years, specifically via the works of Gaudi in the 19th century that Geometry, a key architectural concept, further evolved as the basic tool for bridging the gap between different disciplines related to design and construction. Gaudi advocated the need for geometric rationalization via his experiments with free-form complex shapes. These geometries were difficult to describe using traditional notation systems and hard to analyze structurally, thus evoking innovative solutions via out-of-the-box thinking [5,6].

The beginning of the 20th century saw unprecedented growth in free-form geometry, more specifically in the form of ‘surface structures’ due to the inclusion of reinforced concretes. Traditionally, concretes were mostly used to mimic iron and steel frames to strengthen the structures. With the introduction of reinforced concretes architects were now able to experiment with ‘form-finding qualities of plastic-liquid concrete’ [7]. Thin double-curved concrete surface structures evolved and were frequently used in the works of Candela, Torroja, etc. A very common use-case of these shapes were large dome shapes as they became much more ‘economic’ in terms of material and structural efficiency to cover a large area. At the same time, wood had become equally popular in assisting concrete surface layers. One can find ample use of wood as lightweight construction material in Isler’s designs.

During the same period, fiber-reinforced polymer (FRP) composites were also investigated in detail. FRPs provided various advantages to engineers and architects due to their flexibility, lightweight, and outstanding mechanical properties. They also exhibit properties such as waterproofing and thermal insulation, which made them increasingly popular for use in façade and envelope systems in building constructions. Conventional production of freeform structures with FRP composites involves double curvature grid shells using molds which can vary in terms of scale, material, and complexity [8,9]. Although advantageous, practical prototyping of such free-form shapes is a time and cost-intensive process, and only with recent developments in additive manufacturing and digital fabrication process, various critical issues in the development of such structures are now being answered. These innovations led to architects performing the role of builders once again along with being designers. This helped the constructions to be realized not only in terms of geometry but as well in contrast to frequently occurring constraints, thus resulting in unconventional geometrical constructions.

**Figure 1:**
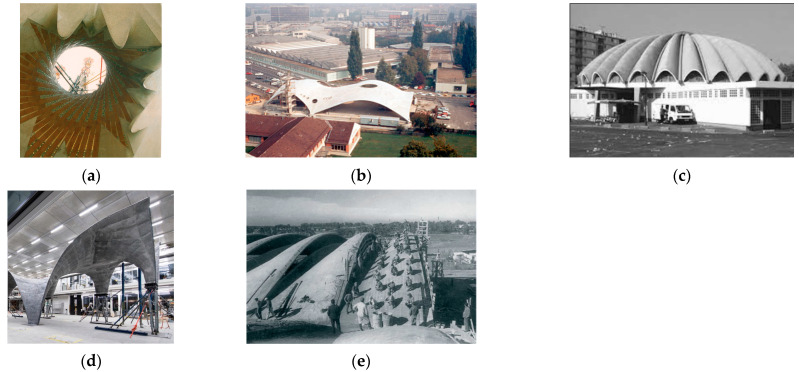
(**a**) Hyperbolic Paraboloids in the interior of the Vaults of “La Sagrada Familía” constructed by Antoni Gaudí, Reprinted with permission from [12]; (**b**) Factory for Sicli SA, Geneva, Switzerland constructed by Heinz Isler [7]; (**c**) Market Hall, Argenteuil, 1967 an example of composite freeform structure created with geometric design approach [8]; (**d**) The full-scale prototype of the roof of the NEST HiLo unit 2017 [13]; (**e**) Dieste’s Gaussian vault under construction. The vault is subject to an ad hoc load test with a distributed load generated by the workforce [7].

In the following years, with the aid of advancements in technology and computational tools, shape realization saw unprecedented growth, which often is termed the digital turn or digital design in architecture. Most notable in this transition phase are the works of Eladio Dieste and Frei Otto. An Uruguayan engineer, Dieste, in his works, emphasized forms being structural elements: “...The resistant virtues of the structures that we seek depend on their form ...There is nothing more noble and elegant from an intellectual viewpoint than this, to resist through form,” thus creating large scale double-curved shapes which could support themselves without the assistance of any ribs or beams [10]. Similar to Deiste and Isler, Otto also considered lightweight constructions and minimalism as the central focus of his research. As an architect, Frei Otto is often considered the designer of membrane and cable net structures. His major contribution to lightweight structures could be his approach to finding self-organized surfaces by virtue of soap experiments [11]. A few examples of famous free-form architectures can be seen in Figure 1.

While these builders emphasized experimental methods, they had a very positive view of the effect of computers and had actively integrated computational methods into their own studies. Digital design (DD), since then, has become synonymous with architectural form-finding, with various sub-categories of computational design (CD) ranging from generative, bio-morphogenetic, parametric, and algorithmic designs, etc., being evolved. In recent years, computer-aided design (CAD) commodities have enabled experimental methodologies to be translated into a virtual domain termed a design space where architects enjoy more freedom to design projects with increasingly complex geometries and abstractions [14]. Some of these shapes have proven difficult and expensive to realize, even using state-of-the-art fabrication processes. Consequently, architects often need to modify the geometries according to the constraints of fabrication. This process of making a complex design feasible for manufacturing within the limitations of available machinery; and affordable as compared to regular design cost by digitally altering its geometry by shape rationalization is termed fabrication-aware design [15].

### 1.3. Computational Design and Visual Programming

As described in the previous section, computational tools and techniques have been an influential part of architectural design in recent decades. The early 1980s saw a revolution in the design process mostly due to the commercial availability of CAD and building information modeling (BIM) tools in parallel with the development of simulation tools, thereby empowering designers to analyze their designs in terms of different performance criteria. Although, these developments were mostly limited to DD. Later, particularly in the 1990s, due to the introduction of automation, the paradigm of CD truly opened up. The main difference between CD and DD is that, while DD is simply the usage of a digital tool to create a geometry in virtual space, CD relies on computation (digital or analog) and need not necessarily bound to exist in digital space. CD, as a design process, takes advantage of existing computational capabilities by virtue of automation, induction, abstraction, parallelization, etc., to provide a more flexible approach to architects and designers in form-finding and mapping simulations. These developments in CD tools have enabled architects to deal with forms that previously could not have been realized and would require non-trivial engineering methods for fabrication [16,17].

**Figure 2:**
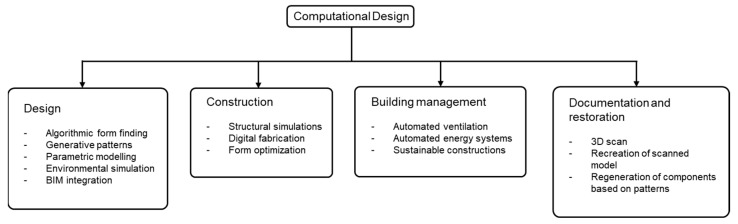
Different phases of CD application in architecture [16,17].

Depending on the nature of the application, the CD can play various roles in construction and architecture (Figure 2), and consequently, a number of computational tools have been developed to perform specific tasks.

There exist major overlaps in the mentioned modules, and as a result, while the possibilities are immense, complexities of translation between different tools also have been increased, thus hindering the process. In this work, we will be focusing more on the design and construction modules of CD and will try to bridge a few aspects of these modules for ease of modeling. The existence and introduction of new design tools are continuously changing the way design is conceived, documented, and represented, and while there exist major overlaps between two sets of form-finding approaches, we still need certain criteria to validate and justify the variety of available solutions. Parametric design thinking is one such approach that relates the solutions to the problem by establishing a temporary geometrical solution space or design space. Thus, a typical parametric design model can be conceived by defining a series of questions to establish the variables of a design and a computational definition that can be utilized to facilitate a variety of outcomes [1,18,19]. Each variable can be set within certain limits, and at any instant of time, a particular set of variables and computational definition would work as the performance criteria for available solutions. The setup can be incorporated with other design models via user-friendly visual programming while hiding complex mathematical definitions behind the interface.

Although parametric thinking and parametric design have been widely adopted approaches in architecture and construction engineering, it is the development in visual programming which really makes it an attractive process for architects to explore alternative design solutions.

Architects primarily are not computer programmers. Moreover, writing computer programs remains one of the most challenging intellectual problems of our age. Visual programming is a tool that makes this process easier using a graphical interface to accommodate smooth interaction between less-trained people and machines. It enables users to describe the process using illustrations with computer language translated into figures and connectors. As studies reflect [20], large groups of people, specifically young students, tend to learn more visually as compared to textual forms. Hence visual programming becomes an easy ‘gateway’ into the programming domain for many creative learners. This allows researchers from different domains to exercise their creativity within the computational domain, with minimal training required.

Although visual programming can simplify complex problems with drag-and-drop components, many challenges arise when the complexity of the visual or graphical script overwhelms the user. Some complex models can consist of hundreds of components that are connected to each other, thus leading to visual entanglement [21]. These problems can be avoided by either keeping the scope of the application at a more introductory and intermediate level or using intermediate visual scripts, which allow users to write custom computer codes, thus reducing the number of components needed for modeling.

**Figure 3:**
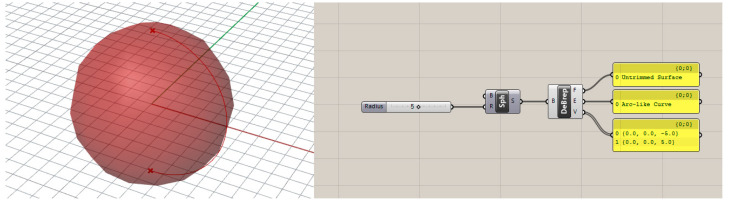
Representative Grasshopper code for a basic parametric sphere with details of various components.

Rhino–Grasshopper [22] is one such visual programming environment that allows users to create their models both via drag and drop method and visual scripting. Figure 3 shows a very basic Grasshopper model for a sphere, the radius of which is parametric, meaning it can be changed as per the requirement of the user. The generated surface can further be exploited using codes to evaluate various other geometric features.

This simple geometry can further be subjected to different other modifications within the Grasshopper domain with the aid of various in-built toolboxes and external plug-ins as per requirements of the design problem.

Key plug-ins and components used in this work include:

- Kangaroo Physics [23] to perform an initial analysis of the evolved forms. It is a Live Physics engine for interactive simulation, form-finding, optimization, and constraint-solving;
- GhPython [24] is a Python interpreter component for Grasshopper to execute custom Python scripts for optimization;
- Galapagos [25] is an evolutionary solver to perform as a validation tool for custom Python programming input;
- Karamba3D [26] is a parametric structural engineering tool for shell analysis;
- Silkworm [27] is a Grasshopper plug-in to translate geometries into customized G-codes.

### 1.4. Biomimicry as a Trade of Design in Architecture

Throughout history, architects and designers have turned to nature as a source of inspiration for different kinds of forms, techniques, and functions. Although it was only by the 1960s that the term Biomimicry, as it is known presently, first appeared in scientific literature and became popular in different streams by 1997 through the lectures of Janine Benyus. The word Biomimicry originates from the Greek terms bios and mimesis. While bios means life, mimesis translates to imitate. Thus, biomimicry in architecture is imitating or copying live nature to achieve sustainable solutions.

This copying from nature can be generally classified into three categories [28,29,30]:

- Organization level: This level of biomimicry is probably the most widely used process where designers mimic the whole or part of a biological form to their benefit;
- Behavior level: At this level, designers observe the behavior of certain biological organisms and try to artificially simulate the same;
- Ecosystem level: This level of mimicking focuses on the functionalities of biological forms and is considered to be the toughest form of mimicking. Adapting biological functionalities implies adapting biological principles and wisdom, which often perform much better than traditional technologies. The application of such wisdom in the form of algorithms has extended the scope of simulation and optimization in architectural models.

For all practical purposes, these three classifications yield significant overlap with each other. For our work, we have adopted a mixed approach of organization and behavioral level biomimicry by mimicking the growth of a plant and adapting it into structural form-finding. For form optimization, we used evolutionary programming, which takes inspiration from the natural selection process and tries to find a global optimum by stochastic search method.

## 2. Materials and Methods

### 2.1. Design Principle

**Figure 4:**
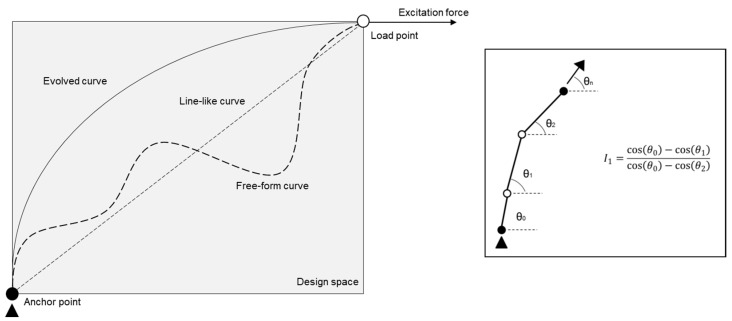
Schematic representation of 2D form-finding process following plant-growth algorithm with representative growth intensity factor for one such instance (in inset).

As described in the previous section, our form-finding approach was to mimic one aspect of the plant growth mechanism, i.e., Phototropism [31], and to create a structural equivalent to it. We took the auxins-light analogy and applied the idea to form-force relationships. We observed that, between two predefined points, a free-form curve, when allowed to self-organize itself following a hyperbolic function, orients itself from the anchor point towards the direction of excitation force, thus resulting in a smooth curvature that exhibits high stiffness under loading in a direction perpendicular to the excitation force. The way this growth mechanism works is that a number of random points are generated between the anchor and load point in the design space at any instance. The points, along with two extremities, perform as control points for an arbitrary NURB curve. This curve is further divided into small line-like segments, and the growth intensity (or the relative difference in direction cosine) of each segment is calculated. A summation of all such values from mentioned segments serves as the fitness of the particular curve. A stochastic evolutionary program takes this value as input and tries to minimize this by changing the randomly generated points. A detailed description of the process and its correlation with other evolutionary solvers is described in our previous work [1], and an illustration of the process is given in Figure 4.

**Figure 5:**
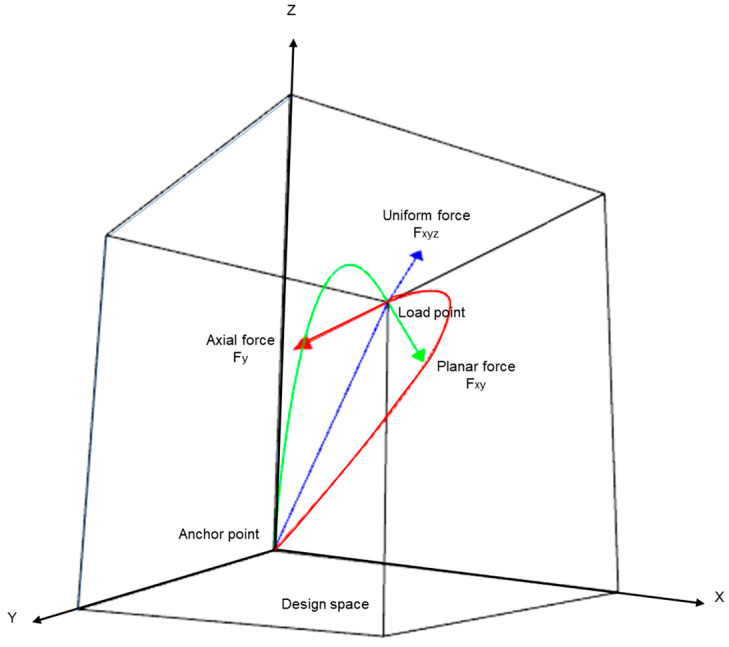
Illustration of 3D design space with evolved curves under different excitation forces with corresponding color codes.

Extending this form-finding approach to 3D design space allows a designer to experiment with different kinds of excitation forces and corresponding forms due to the presence of an additional dimension. Figure 5 briefly describes the process where three variations of forces were taken into consideration. For a force with magnitude equal in all three axes, the optimizer evaluates the solution as a line-like curve, while for a planer force, we obtained a curve that lies on a 2D plane. Thus, the previous example transforms into subsets to the updated system. Interestingly for an axial force, we obtained a curve with a curvature spanning three dimensions.

To investigate the qualities of the curves and to realize them as actual working prototypes, a workflow was devised that undertakes different test surfaces generated from the corresponding curves via different mathematical operators as different case studies, and tries to rationalize them against different loading conditions. The core understanding was that if a curvature exhibits some specific property, it should translate the same while undergoing any transformative operation in generating corresponding surfaces.

Case studies also brought out a series of fundamental questions in linking design and fabrication processes for the construction of the intended shapes. Our key goal here was to establish a parametric workflow that can incorporate various input parameters related to design, structural analysis, and manufacturing unit, keeping ease of usage in mind. Thus, the focus was shifted more to introducing the notion of design integration into the architectural form-finding process via modeling. However, to validate the results, examples were extracted in other structural simulation and external testing domains, and consequent tests were performed. Additionally, key areas of improvement in the workflow were also identified, which can be investigated in the future for further development in the field.

### 2.2. Case Study Preparation

**Figure 6:**
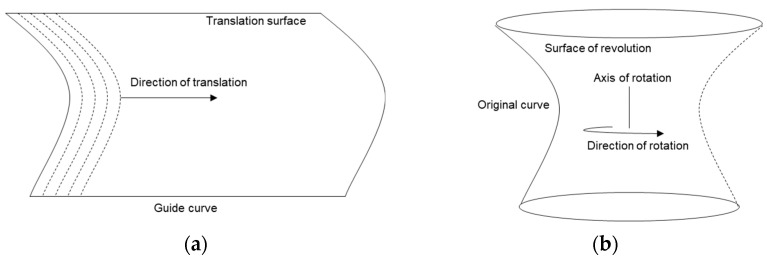
Schematic representation of (**a**) translation surface and (**b**) surface of revolution generated from a curve.

There exist various mathematical operators which transform a curve (or a family of curves) into a surface. Translation surface and surface of revolution are two such cases (Figure 6) that have been used in this work on various occasions.

A curve, when translated parallel to itself via at least another guided curve in such a way that at each point along the second curve, the original is repeated, produces a surface of translation. In contrast, a surface of revolution is a surface in Euclidean space that can be created by rotating a curve around another one as an axis.

**Figure 7:**
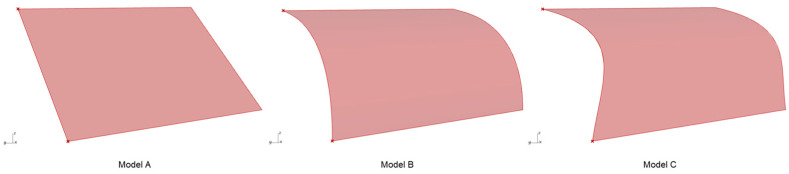
Translation surfaces generated for the first case study.

For design simplicity, the first test case was generated by translating the three generated curves along a line, as shown in Figure 7. For ease of nomenclature, each corresponding model henceforth in this text will be referred to as Model A for shapes generated from a line-like curve, Model B for shapes from a 2D curve, and Model C for shapes originating from a 3D curve.

**Figure 8:**
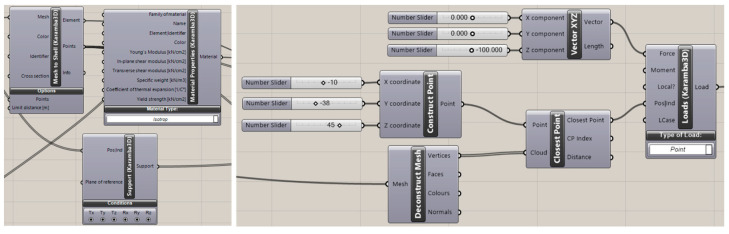
Section of Karamba 3D FE programming.

To establish a shape rationalization process, these models were subjected to various boundary conditions inside Grasshopper parametric modeler using the Karamba 3D plug-in, which helps in calculating the deformation of shell structures using the finite element method. The plug-in offers an extensive data bank for required material properties for both isotropic and anisotropic use-case. For this setup, the material property is kept constant to a specific isotropic value for all the samples. The purpose was to achieve intended relative differentiation among samples based on geometry. Definitions such as boundary conditions, input force information, etc., were kept as parametric inputs to provide flexibility to the setup. As can be seen in Figure 8, in Karamba 3D, any arbitrary loading condition can be set at a random point in space. Node information of a mesh under investigation can be extracted, and loading point information can be mapped on the closest point (s) among the nodes.

**Figure 9:**
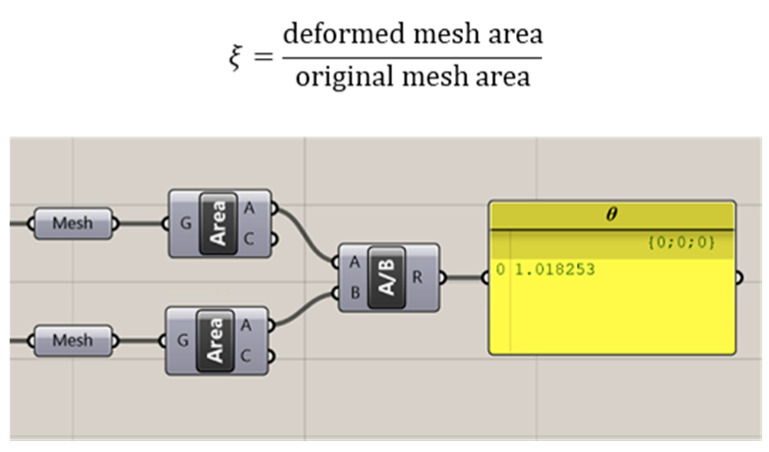
Deformation coefficient *ξ* formulation and corresponding program code.

While such finite element results can showcase stress deformation under different loading conditions, understanding such information would require some level of technical expertise, which might create a roadblock for common users in comprehending different shapes. To ease the process of visualization of the results, we assign a factor *ξ* or deformation coefficient, as shown in Figure 9, which is essentially the ratio between the deformed mesh area after loading to the original mesh area. This gives users an easy and swift overlook of the designed model’s preliminary performances. Once satisfied, the user can further investigate the specific aspects of a shape by changing other parameters.

As exemplars, two specific test cases are mentioned here:

- Random load at a specific point on the surfaces with all four edges being fixed;
- Random point load with extreme two edges of translation being fixed.

**Figure 10:**
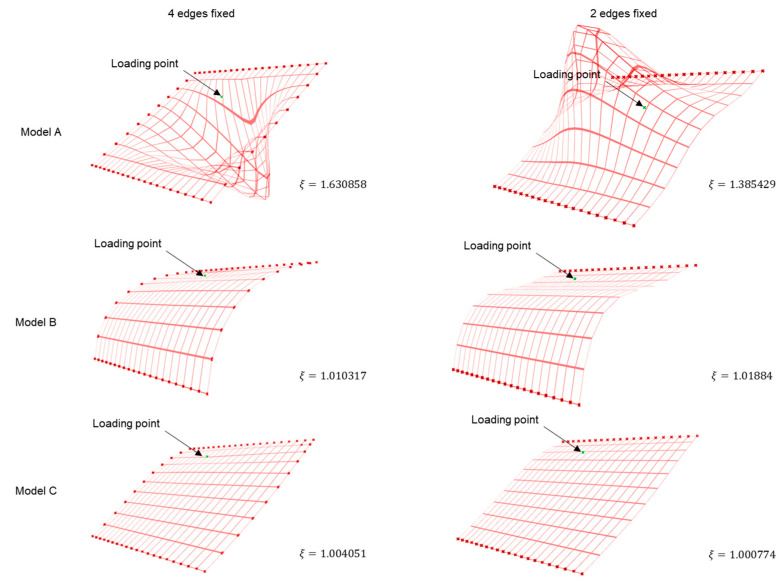
Deformation on grid meshes and corresponding ξ values.

Since the idea here was to rationalize a shape via its geometry, scaled deformation in grid meshes is documented in Figure 10 for better visualization. Moreover, scaled deformations and loading conditions were kept consistent across all models for each test case and hence are not interchangeable across cases. ‘Red cross’ marks represent fixed boundary conditions for each test case, while ‘green cross’ markings represent the location of the unmapped loading point. The attempt here was to establish a baseline of understanding structural deformation via deformation coefficient *ξ* value, and as it can be observed, we achieved a decreasing trend for the same from Model A to Model C within each test case.

### 2.3. Prototyping

**Figure 11:**
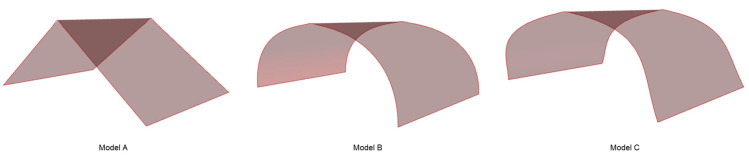
Modified surface models.

While constructions, as described in the previous section, can be realized easily in a design domain, to materialize them into a physical working prototype would require some modification. For ease of construction and testing, a conventional roof or dome-like shape was imagined, which could be easily tested under compressive load. Using mathematical operations described in the previous section, we achieve such shapes, as shown in Figure 11, by joining two axisymmetric surfaces. While the surface generation process is the same as earlier, in this case, we produced surfaces that can be fixed on the ground at two fixed edges, and mechanical tests can be performed on them.

**Figure 12:**
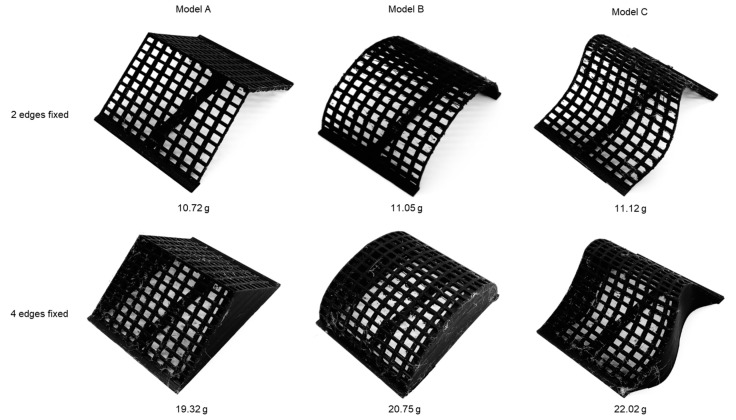
3D-printed prototypes with attachments mimicking boundary conditions.

The first set of prototypes was prepared via standard FDM 3D-printing technology [32]. PLA material [33] was used to create layer-by-layer construction of grid-mesh structures. The shapes were also given additional attachments to imitate necessary boundary conditions, e.g., for compression testing with two fixed sides, a T-shaped attachment was merged with each sample at the bottom in the modeling process itself, and the entire model was printed as a single assembly. A similar approach was followed for compression testing with all fixed sides, where additional side walls were provided to each model in the design process. Finished samples with attachments are documented in Figure 12 with corresponding mass information. The dimensionally bottom area of each sample was fixed as 40 × 56 sq. mm while the maximum height was 20 mm.

While 3D printing enables the realization of the intended shapes, a lot of curvature information is lost in the layer-by-layer manufacturing process constraint. Moreover, to truly realize a 3D free-form shape, anisotropic material behavior needs to be incorporated as part of the prototyping process.

**Figure 13:**
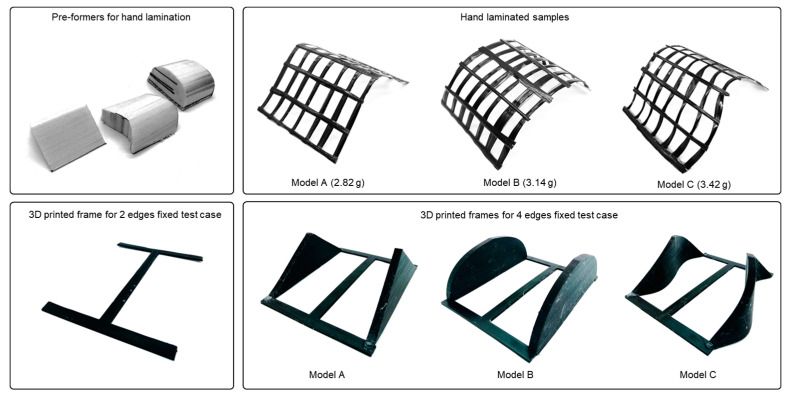
Setup and attachments to prepare and test hand-laminated fiber-grid surface samples.

Thus, in the next step of prototyping, grid models with carbon fibers were prepared via the hand lamination method. External dimensions of the intended samples were kept similar to PLA samples. Corresponding shapes were cut from Styrofoam which worked as pre-formers for this process. Thin sheets of plastic films were cut and fixed on the surfaces of the pre-formers, and grid geometries were mapped on the films. Surfaces of the films were coated with a thin layer of epoxy resin (RIMR 135, Hexion, Duisburg, Germany) to stick fiber filaments (linear mass density of 0.82 g/m) along the mapped grids, and later, each surface was coated with another layer of resin. Finished assemblies were cured at 60° Celsius overnight, and attachments were removed from the fiber grids. To realize the applied boundary conditions, customized 3D-printed parts were prepared for each surface, and for each test case, the corresponding surface was attached to the 3D-printed part, and the setup was subjected to compressive load testing. The preparation setup with individual sample mass is described in Figure 13.

Although the hand lamination process provides a simple process of fiber composite manufacture, the method itself is vulnerable to various unavoidable precision errors and cannot serve as a manufacturing process for high-performance composite systems.

To address this issue, the setup was transferred to the standard VARI process, where fibers are infused with a resin system in the presence of a vacuum. Scaled-up samples with a base area of 100 × 140 sq. mm and a maximum height of 80 mm were conceptualized in this process as the last set of prototypes. The samples were further modified to have elongated horizontal lengths through which resins can enter and exit the system easily. Furthermore, two-part molds were created for each surface such that fibers can be easily placed between them along designated grooves, and the parts can be joined together afterward. Scaling-up also helped in increasing the number of grooves for fibers to achieve condensed surface meshes. Once modeled, the parts were transferred to an FDM printer, and water-soluble PVA material [34] was used to print the mold parts.

**Figure 14:**
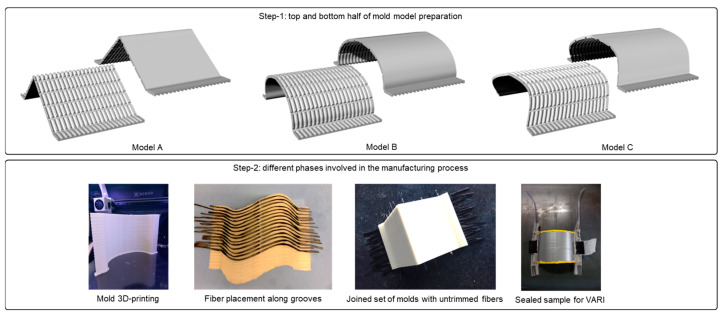
Illustration of different steps involved in the mold-assisted VARI process.

**Figure 15:**
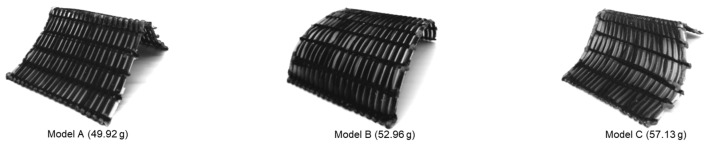
Final cleaned and trimmed carbon fiber composite samples.

The standard wood spray was used to place and temporarily attach fibers on specific slots in the printed parts, and once the process was complete, additional fibers were trimmed to have clean inlets and outlets on the mold surfaces. Prepared molds were then sealed properly, and vacuum bags were used to infuse resin through the desired channels. The complete setup was then cured at 80° Celsius with additional supports below the surfaces to avoid any deformation transferring from the mold surface to the fibers inside due to the high temperature. Different phases involved in the manufacturing process are summarized in Figure 14. Once cured, the molds were submerged in a water bath for 24 h to be fully dissolved. Samples were then cleaned, and additional attachments were trimmed away, as can be seen in Figure 15.

## 3. Results and Discussions

### 3.1. Test Setup

**Figure 16:**
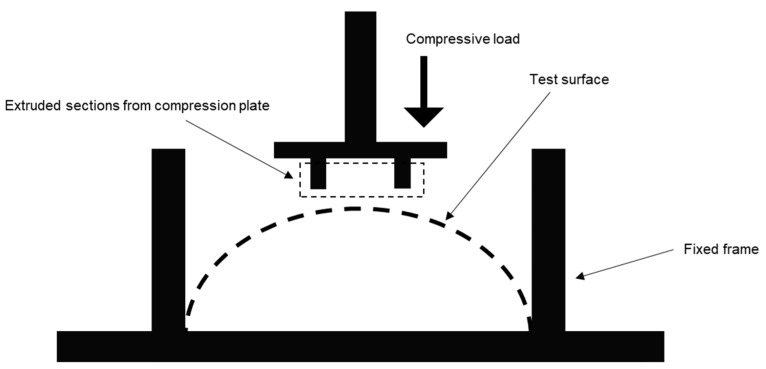
Schematic representation of the test setup.

Once prepared, all prototypes were subjected to a compressive load test, where a probe was slowly pressed against the surfaces of the samples until the breaking point while the bottoms were fixed. The probes were designed such that two extruded sections come in contact with the test surface at any point. This was to avoid transmission of the load directly at the mid-section of the surfaces, as the purpose was to calculate load bearing capacity of the entire surface. As described in the previous section, both for PLA and hand-laminated prototypes, boundary conditions were realized via 3D-printed attachments. For VARI samples, the setup comprised two metal blocks pressing the test surfaces from two opposite sides to realize one set of boundary conditions, as can be seen in Figure 16. Due to logistic limitations, such testing for test cases with all fixed boundaries was avoided for these samples.

### 3.2. Results

**Figure 17:**
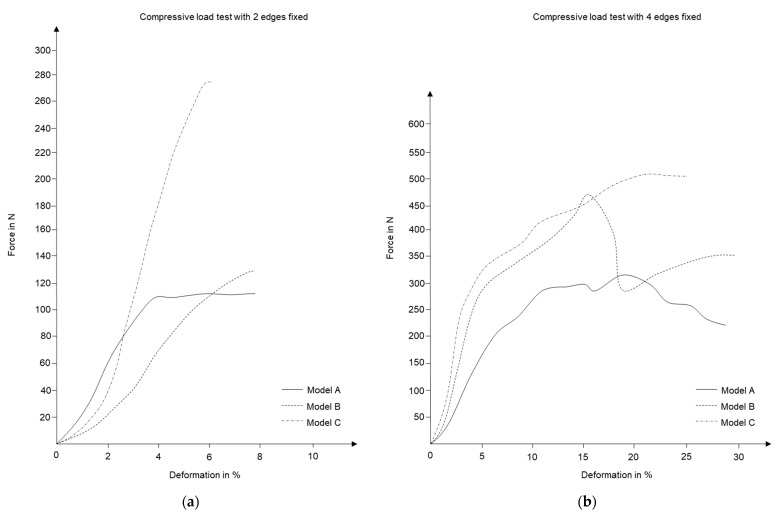
Compressive load test results for 3D-printed surfaces with (**a**) two edges of translation being fixed, (**b**) all open boundaries being fixed.

Figure 17 represents the comparative test results of 3D-printed samples. As can be seen, the performance quality of Model C is superior under compressive load for both types of boundary conditions. With two edges being fixed, it can sustain almost double the load as compared to the other two surfaces. The scale of comparison decreases with four edges being fixed. This probably is due to additional support on the sides, which strengthens all test samples.

**Figure 18:**
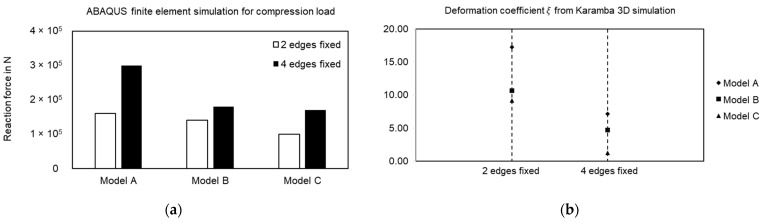
(**a**) Compressive point load simulation on surface models in ABAQUS, (**b**) deformation coefficient 𝜉 value comparison for random point load simulated in parametric solver.

To validate the results, a similar setup was replicated in ABAQUS [35] finite element simulation domain along with Karamba 3D analysis with deformation coefficient value 𝜉. Figure 18 summarizes the results from both simulation platforms. While in each of the above-mentioned cases, loading values differ, they all have consistent boundary conditions, and hence the trends in relevant criteria among the samples should be comparable.

In ABAQUS analysis (Figure 18a), for a representative compressive load of 100 N, the reaction force generated in the system decreases from Model A to Model C for both boundary conditions under consideration. A low reaction force implies that the system undergoes less deformation compared to others under loading, reflecting a higher stiffness value. Moreover, for the case with two edges being fixed, the gap between Model C and other two samples are large, while the gap reduces in other case with Model B. These results closely replicate the findings from experiments and the trends from Karamba 3D analysis (Figure 18b) and serve as the initial proof of concept.

**Figure 19:**
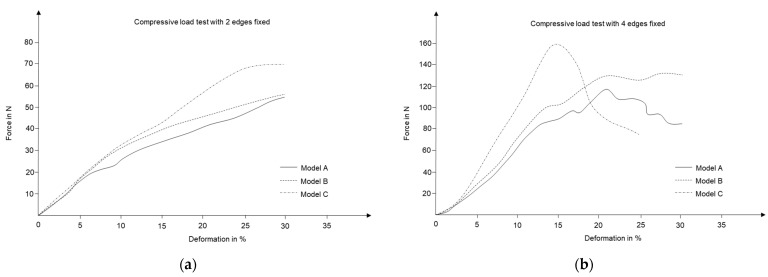
Compressive load test results for hand-laminated fiber-grid surfaces with (**a**) two edges of translation being fixed, (**b**) all open boundaries being fixed.

**Figure 20:**
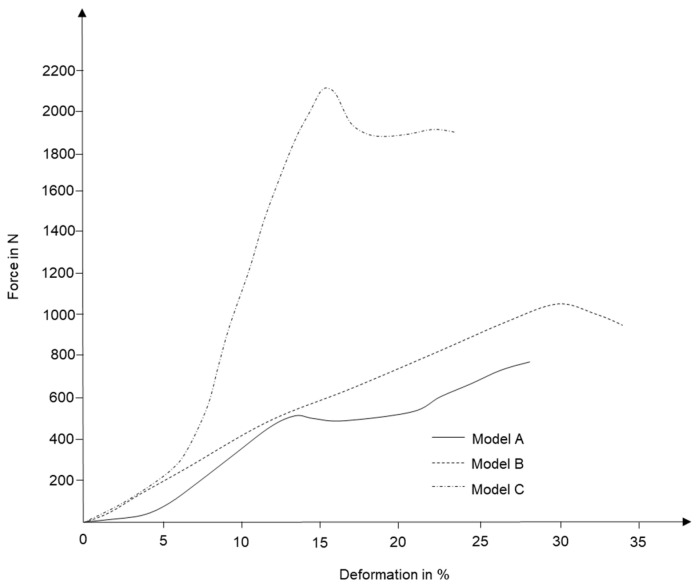
Compressive load test results for fiber composite samples with two edges of translation being fixed.

The next step was to realize the same process with fiber composite samples. For this purpose, both hand-laminated samples and VARI samples were subjected to compressive loads. Figure 19 demonstrates results from hand-laminated samples, while Figure 20 represents test results from VARI samples.

The resulting trends are in contrasting agreement with previous test cases, and it can be easily argued that integrating 3D curvature within a geometry not only improves the performance of a shape but also assists in elevating the anisotropic material behavior of construction elements.

### 3.3. Additional Results

**Figure 21:**
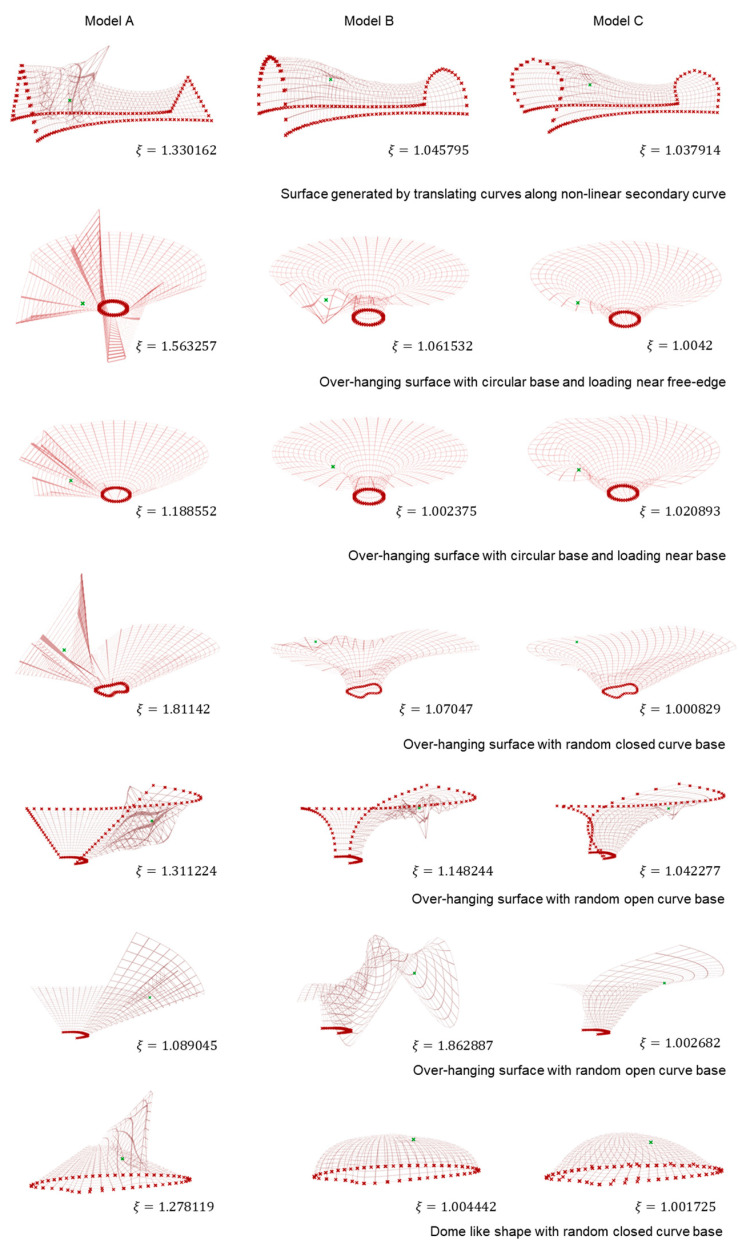
Deformation illustration of different surfaces generated from basic curves along with their *ξ* values under different boundary and loading conditions.

In the previous section, we tried to devise a workflow to generate, fabricate and test simple surface structures using fiber composites. Moreover, we also established that deformation coefficients could safely predict the nature of the generated surfaces to provide a rough initial idea. As prototyping is a complex process, the case studies were limited to only linear translation surfaces. However, in the design domain, various such cases can be conceptualized. A few such cases are illustrated in Figure 21 with corresponding deformation coefficients.

In each test case, boundary conditions are displayed as ‘red cross’ symbols while random loading points are shown as ‘green cross’ symbols. For better visualization, deformation in grid-meshes is mentioned here instead of entire surface analysis, and deformations are scaled to fit the visualization window.

It is interesting to note that based only on surface analysis, few key information can be extracted for such shapes:

- For closed boundary conditions, shapes generated based on 3D curvature curves perform better;
- In the case of free-edge deformation, loading conditions play a key role in deciding the hierarchy of the solutions. For disturbances near open edges, models generated based on 3D curvature curves are preferable solutions, while disturbances near the base would be better encountered by shapes generated from curves with 2D curvature;
- With parametric modeling, it is easy to generate and modify surfaces. However, with each modification, new boundary conditions are introduced in the system, which needs to be considered in the analysis section.

### 3.4. Preparation for Digital Fabrication

In previous sections, we described a few ways to prototype free-form shapes using conventional manufacturing techniques. While the methods described in the work provide sufficient information to validate results obtained via parametric workflow, to truly realize free-form shapes, design space is needed to be integrated with the digital fabrication process.

Digital fabrication in architecture and engineering is a relatively recent phenomenon, emerging over the last couple of decades to become a substantial aspect of critical debate, professional practice, and education within the discipline. Essentially, digital fabrication is a sub-category of Computer-Aided Design and Computer-Aided Manufacturing (CAD/CAM), bridging the gap between these two domains by utilizing computer-controlled machines as tools to cut or make parts.

In CAD/CAM process, engineers design and develop components within three-dimensional modeling software and then convert the models into Geometric code (G-code). G-codes are digital information of the designed geometry stored as the collection of coordinates in space which in turn can be used as instructions for the tool paths in rapid prototyping processes, thus translating digital design into physical reality. This process of interaction enabled greater fluidity between design generation, development, and fabrication compared to traditional approaches, which necessitated a more cumulative, staged process. This ease in the interaction between the initial and final phases enabled architects to participate more in past decades, thus pushing the boundaries of form generation and construction [36,37].

**Figure 22:**
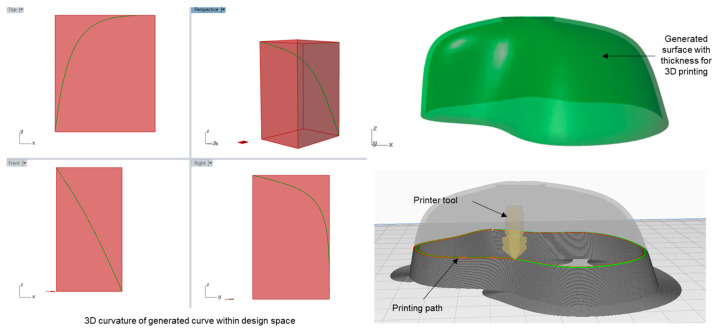
Illustration of layer-by-layer slicing in conventional 3D printing slicer Ultimaker Cura [38] and loss of geometry definition. Here a geometry created from a 3D curvature (denotated by green curve on left) is reduced to 2D definition (denoted by printing path on right) due to printer limitation.

As discussed earlier, the layer-by-layer approach of the conventional additive manufacturing process has limitations in that they redefine the curvature property of the intended geometry, as can be seen in Figure 22. Thus, while they produce acceptable outcomes with isotropic materials, the use cases are limited for anisotropic materials such as long fibers.

**Figure 23:**
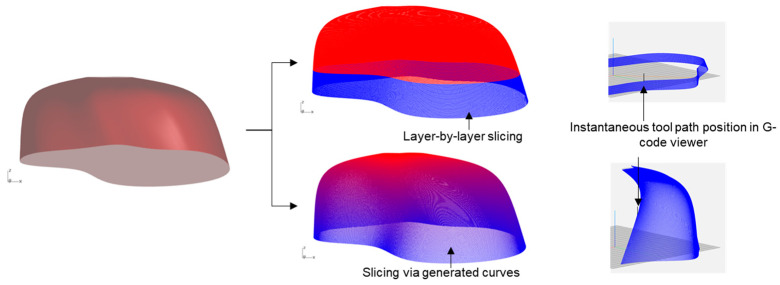
Two different slicing processes using Silkworm toolbox and corresponding G-code display in NC Viewer [39].

Our attempt here was to address this issue by customizing the G-code, which can be translated to a robotic arm, thus creating an in-house framework for the next-generation digital fabrication process. For this purpose, we used Silkworm, a Grasshopper toolbox that enables designers to slice the geometry inside parametric modeling itself. Instead of the default slicing mechanism, which would yield layer-by-layer G-code, we deconstruct the generated surface in terms of evolved curves. Thus, instead of slicing the surface, Silkworm would take multiple curves as user input and result in the desired G-code tracing of those curves. To accommodate any geometric constraint, which would arise due to collision between the tool path and printed object, the number of array curves was taken as parametric input. Figure 23 illustrates two different ways Silkworm slices the same geometry in the design space.

**Figure 24:**
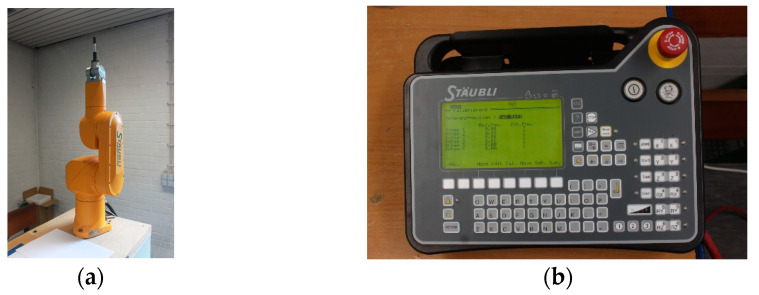
(**a**) Robot arm with attached marker, (**b**) manual controller.

Once the desired G-code was obtained, the next step was to develop a process that translates generated G-code into a language that is understandable for the robot arm. To achieve this, the target system was first examined, and robot movements were confined by the manual control unit. The in-house setup of Stäubli RX60 and the controller is shown in Figure 24.

**Figure 25:**
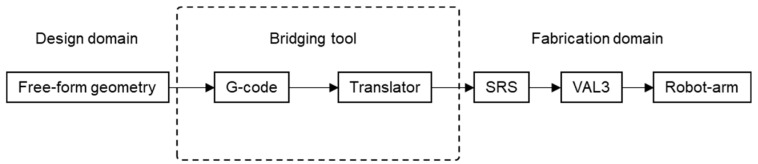
Flowchart showing digital fabrication process.

VAL 3 [40] is the program embedded in the robot arm. To establish communication with the robot arm, we used an in-house translator built to convert user input G-codes required language. Stäubli Robotics Suite (SRS) [41] was used to emulate collision control between the robot arm and the intended shape in a virtual PC environment. This serves as a feedback mechanism to resolve the conflict between the design and fabrication domains. Thus, a flowchart, as shown in Figure 25, was created, which connects all the building blocks for the final information flow from the digital to the fabrication domain.

**Figure 26:**
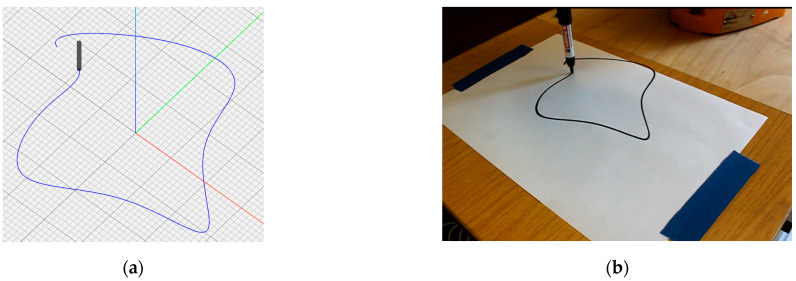
2D shape visualization with (**a**) G-code information display in NC viewer and (**b**) curve drawn by a robot arm with same information as input.

**Figure 27:**
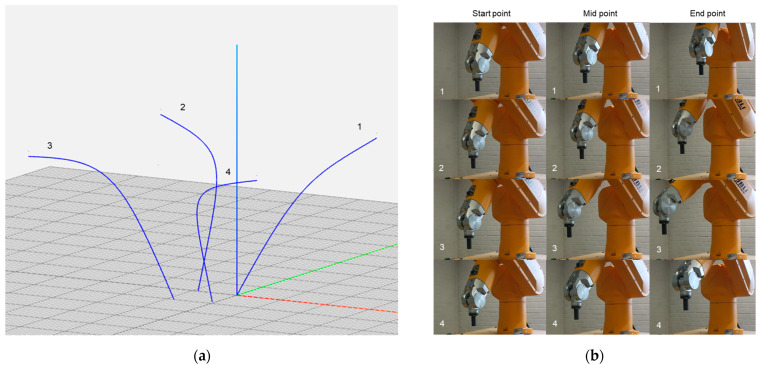
3D shape visualization of four structural members of a system identifiable as 1,2,3 and 4 with (**a**) G-code information display in NC viewer and (**b**) corresponding start, mid, and end frames of robot arm movement for each corresponding curve.

As the main focus of this work was to prepare the framework for the design aspect of the intended process, manufacturing with actual material was beyond the scope and would require different expertise. Thus, to visualize the working of the setup, a few adjustments were made. For 2D curve visualization, a pen tool was attached to the end of the robot arm, which can draw the shapes according to the user’s instruction, as can be seen in Figure 26. For 3D visualization, videos of the robot arm movement were captured, which returned satisfactory results with reference to designed curves. In Figure 27, we tried to explain the correlation by describing screenshots for each member of the geometry at the start, mid and end points of the tracked path.

## 4. Conclusions

A process of shape rationalization for the free-form surface structure was discussed using biomimicry as a prime source of inspiration. We took inspiration from the growth of a plant and applied it to exploit the force-form relationship. We applied evolutionary programming to optimize the process and created an interactive geometrical model curve that reacts to the excitation force. These models perform as the fundamental building blocks for our intended free-form shapes, and in this work, we chose the use-case of a simple dome-like surface undergoing compressive loads under different boundary conditions. Using various toolboxes inside Grasshopper visual programming environment, we created a workflow that takes initial curves as inputs and as outputs, generates surface geometries, provides initial structural deformation assessments, and prepares the geometries for fabrication. For the analysis section, the goal was to ease the process of understanding structural deformations. Thus, a deformation coefficient factor 𝜉 was introduced, which essentially is the ratio of the deformed mesh area to the original mesh area inside the parametric model, and provides a rough preliminary estimation of the performance of a shape.

Moreover, generated models were 3D printed and tested outside the digital space with validation methods providing good correlations with preliminary observations. For fiber-reinforced composite prototypes, we adopted two approaches. The most basic hand-laminated composite provided a basic understanding of dealing with fiber filaments. The initial results of the compression test on the samples were encouraging. Thus, for the next step, we opted for a novel VARI process using 3D-printed molds. Water-soluble 3D-printed molds were manufactured, and fiber filaments placed along the grooves within the molds were infused with resin in the presence of a vacuum. Compressive load tests performed on the composite surfaces reflect close resemblance with other sets of results and thus validate the assumption that, for a given external condition, a shape following 3D curvature would perform superior as compared to more traditional line-like or 2D curvature geometries. A few other test cases were also briefly discussed to elaborate on this point. It can also be observed that the model generated from 3D curvature is slightly on the heavier side in each use case. Thus, it can also be argued that these samples exhibit higher strength due to the presence of additional material. Hence, for lightweight constructions, this can be valuable information to know how to adjust material in space to achieve a stronger shape.

Using parametric modeling, we were also able to transform the whole process into input requirements (as boundary constrain) and obtained geometry output (as G-code data). Digital fabrication essentially is the integration of geometrical data generation workflow inside the design domain with the corresponding manufacturing process. Here we tried to achieve this by translating generated G-code data to a 6-axis robot arm via an encoder. The preliminary results were satisfactory. Although, a more complex geometrical shape would require further improvements in the manufacturing process.

## Data Availability

The data presented in this study are available on request from the corresponding author.
